# The Role of Protein–Lipid Interactions in Priming the Bacterial Translocon

**DOI:** 10.3390/membranes14120249

**Published:** 2024-11-24

**Authors:** Matt Sinclair, Emad Tajkhorshid

**Affiliations:** 1 Department of Biochemistry, University of Illinois Urbana, Champaign, IL 61801, USA; mts7@illinois.edu; 2 Theoretical and Computational Biophysics Group, NIH Resource for Macromolecular Modeling and Visualization, Beckman Institute for Advanced Science and Technology, University of Illinois Urbana, Champaign, IL 61801, USA; 3 Center for Biophysics and Quantitative Biology, University of Illinois Urbana, Champaign, IL 61801, USA

**Keywords:** molecular dynamics, translocase, insertase, protein–lipid interactions

## Abstract

Protein–lipid interactions demonstrate important regulatory roles in the function of membrane proteins. Nevertheless, due to the semi-liquid nature and heterogeneity of biological membranes, and dissecting the details of such interactions at high resolutions continues to pose a major challenge to experimental biophysical techniques. Computational techniques such as molecular dynamics (MD) offer an alternative approach with both temporally and spatially high resolutions. Here, we present an extensive series of MD simulations focused on the inner membrane protein YidC (PDB: 6AL2) from *Escherichia coli*, a key insertase responsible for the integration and folding of membrane proteins. Notably, we observed rare lipid fenestration events, where lipids fully penetrate the vestibule of YidC, providing new insights into the lipid-mediated regulation of protein insertion mechanisms. Our findings highlight the direct involvement of lipids in modulating the greasy slide of YidC and suggest that lipids enhance the local flexibility of the C1 domain, which is crucial for recruiting substrate peptides. These results contribute to a deeper understanding of how protein–lipid interactions facilitate the functional dynamics of membrane protein insertases, with implications for broader studies of membrane protein biology.

## 1. Introduction

Cellular membranes serve a myriad of functions in biology beyond encapsulating cells and organelles, including signaling, regulation, metabolism, and material transport [1]. Many of these functions are facilitated by membrane proteins, which are embedded into the membrane with the aid of a variety of insertion machineries [2,3,4,5]. Examples of recent efforts to characterize the insertion of proteins by members of each family of insertion proteins include SecYEG [6,7,8,9], twin arginine translocase (TAT) [10], and YidC [11,12,13,14,15]. Among these, YidC has garnered particular attention due to its unique mechanism of inserting small membrane proteins independently, despite being a single-chain insertase.

YidC and its homologs, Oxa1 and Alb3, are crucial membrane insertases found ubiquitously in all kingdoms of life, located in bacterial, mitochondrial, and thylakoidal membranes [2]. YidC aids in insertion and chaperoning of ribosomally nascent peptides into the membrane both independently [13] and cooperatively with the well-studied SecYEG complex [8]. It has been shown that YidC is necessary for growth in *E. coli* [16] as well as for envelope biogenesis and biofilm formation in *S. mutans* [17]. As such, it constitutes an attractive therapeutic target, and there is a need for a more complete understanding of its dynamics and function [18].

Previous research has established several key features of the structure and dynamics of YidC. The Dalbey research group demonstrated that water accessibility of the transmembrane pore is limited to the cytoplasmic end of the pore [19]. Meanwhile, past molecular dynamics simulations reported local membrane clamping of the TM domain [20]. The critical role of the C1 domain, which is a coiled coil that sits along the headgroup plane of the cytosolic leaflet, in substrate insertion was clarified using the *B. halodurans* isoform [21]. Finally, the stabilization of the C1 domain by the C2 loop has been demonstrated in several recent publications [22,23]. Recent crystallography studies on YidC have resolved the most complete experimental model of the protein, leading to a renewed interest in the *E. coli* isoform (RCSB: 6AL2) [24].

Despite the availability of this structure, there has yet to be any examination of the role the local lipid environment plays in YidC’s structure or dynamics. Protein–lipid interactions have long been thought critical for the recruitment of peripheral membrane proteins [25,26]. Recent advances in structural biology have enabled the study of such interactions in integral membrane proteins as well [27,28,29,30]. Simultaneously, molecular simulations have continued to elucidate the role of protein–lipid interactions in such membrane-embedded proteins [31,32,33,34,35,36].

In parallel to the advances in structural determination of YidC, recent developments in the study of membrane lipid composition have yielded increasingly higher-resolution membrane models [37,38,39,40]. Computational bacterial membrane models historically consist of purely POPE (1-palmitoyl-2-oleoyl-*sn*-glycero-3-phosphoethanolamine) [41] or a 3:1 ratio of POPE/POPG (1-palmitoyl-2-oleoyl-*sn*-glycero-3-phosphoglycerol) [42] in order to coarsely represent the headgroup diversity found in many bacterial species. Since the inner membrane composition of *E. coli* was first reported in 2006, realistic membrane models have been attracting considerable interest [43]. Leveraging agreement in the data from two different mass spectrometry experiments [43,44], the Top6 membrane model [37,38] was built, which captures not only the headgroup diversity but also the variety of lipid acyl tail lengths and moieties found in *E. coli*.

It is now established that protein–lipid interactions play a role in both the structure and dynamics of many membrane-embedded proteins [45]. However, the influence of native lipids on the YidC family of protein insertases remains largely unclear. By incorporating both the most complete structure of YidC in *E. coli* with a realistic membrane, we have generated an extensive set of simulations which both recapitulate previously reported behavior and highlight new protein–lipid interactions. In doing so, we have obtained further insights into how lipids may potentially aid in the insertion mechanism of YidC, which is complementary to current models in the field [14].

## 2. Methods

**System Preparation.** The most complete experimentally derived *E. coli* YidC crystal structure (PDB ID:6AL2, Chain A, 2.8 Å) [24] was selected for the MD simulations. This model is missing 48 residues at the N-terminus representing a transmembrane helix and linker to the periplasmic domain as well as 16 C-terminal residues. Crystallographic water molecules and the co-crystallized lipid (2R)-2,3-dihydroxypropyl-(9Z)-octadec-9-enoate (OLC) were discarded. Three different lipid environments were selected for simulation in order of increasing complexity to ensure that observed protein dynamics were decoupled from the choice of membrane. To this end, one replica each was generated consisting of (1) pure POPE, (2) a 3:1 mixture of POPE/POPG, and (3) the Top6 membrane as described by the Klauda group [37,38]. The Top6 membrane was developed to mimic the lipid acyl tail heterogeneity of the *E. coli* inner membrane as identified separately by two mass spectrometry studies [43,44]. Besides POPE, the Top6 membrane also contains the lipids PMPE (1-palmitoyl-2-cis-9,10-methylene-hexadecanoic-acid-sn-glycero-3-phosphoethanolamine), QMPE (1-pentadecanoyl-2-cis-9,10-methylene-hexadecanoic-acid-sn-glycero-3-phosphoethanolamine), OYPE (1-oleoyl-2-palmitoleoyl-sn-glycero-3-phosphoethanolamine), PMPG (1-palmitoyl-2-cis-9,10-methylene-hexadecanoic-acid-sn-glycero-3-phosphoglycerol), and PYPG (1-palmitoyl-2-palmitoleoyl-sn-glycero-3-phosphoglycerol). The distribution of lipids in each leaflet of the Top6 replica can be found in Table 1. This slight imbalance in lipid number from the upper versus lower leaflets is due to how the large periplasmic domain of YidC sits on the upper leaflet of the membrane, decreasing the available surface area.

The 3 different replica types were all generated using the Bilayer Builder in CHARMM-GUI and the structure of YidC from the Orientation of Proteins in Membranes (OPM) database to ensure that the orientation relative to the membrane was consistent with expectation [46,47,48,49,50,51,52]. Each replica was solvated with TIP3P water [53] and neutralized with KCl to a total ion concentration of 0.15 M. The resulting systems were all of size 155 × 155 × 150 Å which comprises about 300,000 atoms.

From these 3 different systems, additional replicas were generated using the new Membrane Mixer tool in VMD [54,55]. Using this tool, 50% of all lipids in both leaflets were shuffled to ensure that the initial lipid environment did not bias results, which was particularly important in the Top6 membrane replicas. We generated 3 additional replicas of the POPE system, 4 replicas of the POPE/POPG system, and 7 replicas of the Top6 system for a total of 14 replicas. We chose to sample the Top6 system more heavily as it is a more detailed representation of the native environment of the protein.

**Simulation Protocols.** All MD simulations were carried out with NAMD3.0 [56,57] using the CHARMM36 force field to model all molecules in the system [48,49,58]. The particle mesh Ewald (PME) method [59] was used for long-range electrostatic interaction calculations every 4 fs. Non-bonded interactions were calculated with a smoothing function beginning at 10 Å and a cutoff of 12 Å. Pressure was held constant at 1 atm using the Nosé–Hoover Langevin piston algorithm [60,61]. Temperature was held constant at 310 K using Langevin dynamics with a 1 ps^−1^ dampening coefficient. All covalent bonds with a hydrogen and the H-O-H water bond angle were fixed using the SETTLE algorithm [62]. The simulation timestep was set to 2 fs for the course of the equilibration as well as the production MD.

Each system was first treated with energy minimization for 10,000 steps using the steepest descent algorithm. Afterwards, the alpha carbons of the protein and heavy atoms of the lipid headgroups were harmonically restrained (*k* = 5 kcal/mol/Å^2^) during a gentle heating scheme that consisted of incrementing the temperature by 1 Kelvin every 10 steps until the target temperature of 310 K was reached. The restraints were continued for 5 ns at this temperature before relaxing the lipid headgroup harmonic restraints. The system was then equilibrated with just protein heavy atoms restrained for 10 ns. After this first phase of equilibration, the lipid tails were appropriately melted, and the protein restraints were relaxed and the system allowed to equilibrate for a further 15 ns unrestrained. Each system was then simulated for 500 ns for a total of 7 µs of sampling across all replicates.

All analysis was performed using VMD [54] and its suite of built-in tools, in addition to custom scripts in TCL/Python. Kernel density estimation was performed using the Seaborn plotting package in Python, which leverages a Gaussian kernel density estimation function from the SciPy library [63,64]. All renderings were produced using the Molecular Nodes extension in the 3D rendering software Blender [65,66].

## 3. Results

### 3.1. Top6 Replicas Recapitulate Bulk Membrane Features

**Figure 1:**
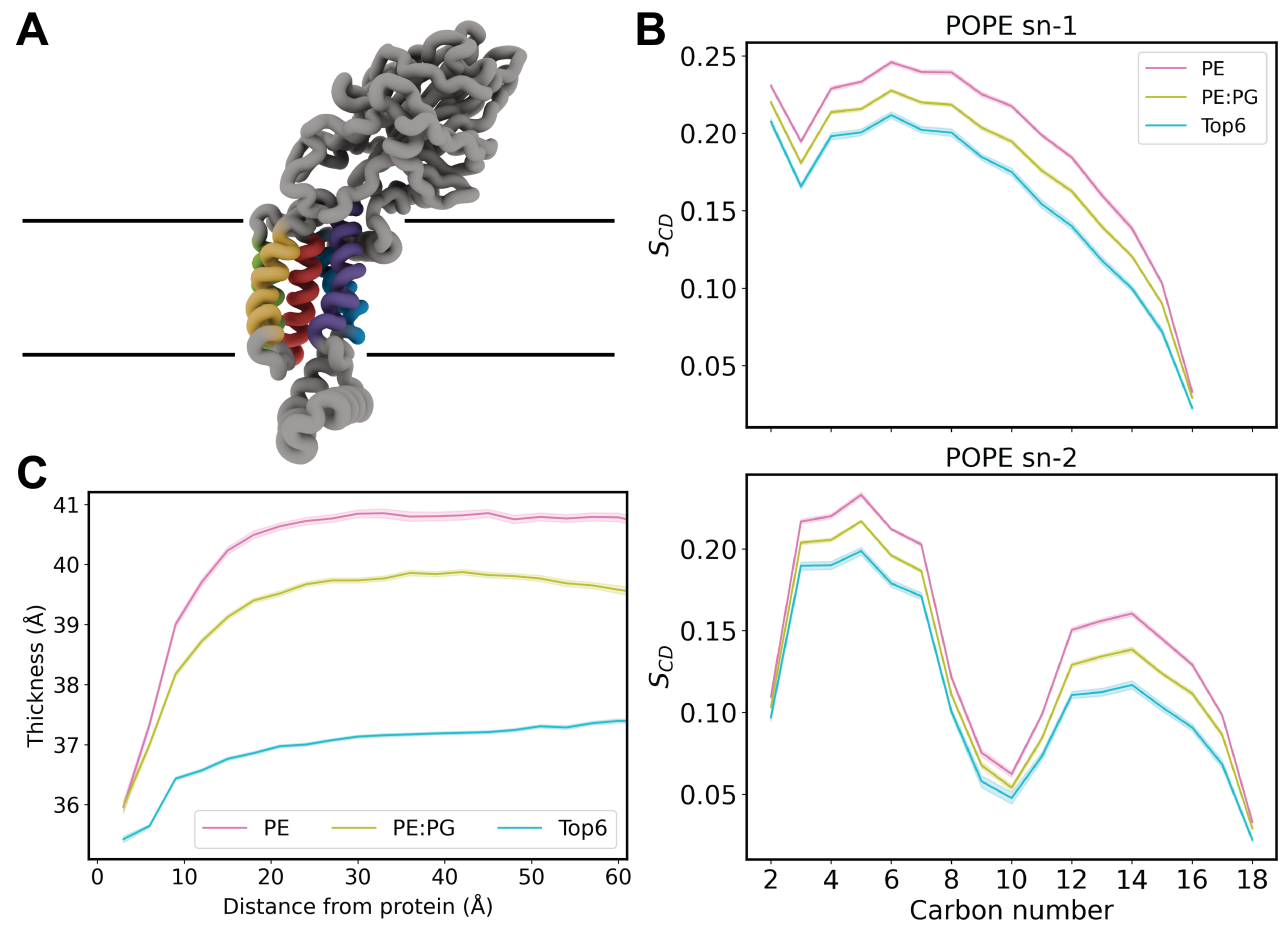
(**A**) Representative snapshot of YidC embedded in the membrane. TM helices 2-6 are represented in blue, purple, green, yellow, and red, respectively. Approximate membrane position is indicated by horizontal lines. (**B**) Deuterium order parameters calculated for POPE lipids in each simulation type for both SN1 and SN2 acyl tails. These measurements were made using the MEMBPLUGIN tool for VMD [69]. (**C**) Membrane thickness measured radially from protein transmembrane helices.

Our first aim was to evaluate the agreement between our system and bulk behavior of past MD studies on YidC [19,20]. A snapshot of YidC relaxed in the Top6 membrane (Figure 1A) highlights the five resolved transmembrane (TM) helices, numbered 2 through 6. The missing TM helix 1 is thought to be an uncleaved signal peptide and may bind to the co-chaperone complex SecYEG [67] but has yet to be resolved experimentally. The lack of TM helix 1 has additionally been shown to be a conditional defect, but it is not essential for viability of *E. coli* [21,68].

Despite the differential membrane composition in our Top6 replicas, we recapitulated local membrane thinning of YidC also previously reported [19,20] as well as trends in deuterium order parameters [37,38] for POPE lipids. Membrane thickness (Figure 1B) was measured as the average *z*-distance between phosphorous atoms of each leaflet radially in discrete 3-Å rings from the TM domains. Long-distance measurements illustrate the bulk membrane thickness, while measurements made within 10 Å capture local membrane clamping that has been previously observed in YidC simulation in mixed PE/PG bilayers [19,20]. Despite being thinner membranes, the Top6 systems also recapitulate this clamping behavior, albeit less dramatic than previously reported.

To compare the rigidity across the various membrane types, we performed deuterium order parameter (S_*CD*_) calculations (Figure 1C). These S_*CD*_ calculations were performed for the POPE lipid only, as it is the sole lipid common in all membrane compositions sampled. The above reported values are consistent with previously reported trends in deuterium order parameters measured in membrane-only simulations [37,38]. The increase in variance for Top6 membranes aligns with the relatively low abundance of POPE lipids in Top6 systems and systems with a smaller patch size than bilayer-only systems used in past studies. These measurements highlight the effect that bulk lipid acyl tail length and unsaturation have on the order of bilayers and subsequently membrane-embedded proteins.

### 3.2. Divergence in RMSD Suggests TM Helices Are Dynamic

**Figure 2:**
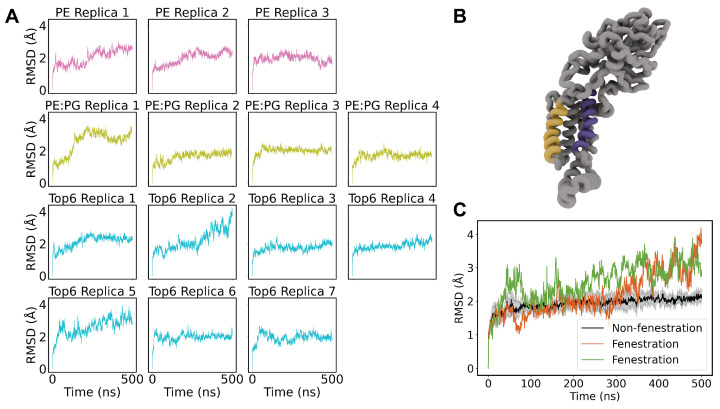
(**A**) Backbone RMSD of TM helices for each system. The images are colored by membrane composition where pink is pure POPE, green is POPE/POPG, and blue is the Top6 membrane. There is a clear convergence in most replicas of approximately 1.5 Å with Top6 replicas 2 and 5 being notable exceptions. (**B**) Structure of YidC, highlighting TM3 (purple) and TM5 (yellow), known as the greasy slide. (**C**) Backbone RMSD of TM helices for Top6 membrane systems. The five converged replicates are aggregated with the mean in black and standard deviation shown in shaded gray. The two divergent replicates are shown as colored traces.

To further assess the health of our systems, we performed root-mean squared distance (RMSD) calculations on the TM helices of YidC (Figure 2A). We chose to exclude the cytosolic C1 and C2 loops, as well as the large periplasmic domain, in order to more clearly assess how the protein is sitting in the membrane. Separate RMSD calculations were performed for the periplasmic and cytosolic domains, which did not indicate any notable dynamics or divergence from replica to replica.

As expected, most replicas of each membrane composition showed convergence, implying relaxed equilibrium systems. Five replicas of the Top6 composition converged to an RMSD of about 1.5 Å; however, two stand-out replicas showed divergences in this RMSD (replicas 2 and 5). While most replica simulations demonstrate some helical rearrangement, most relaxed within about 100 ns, while the two divergent replicas demonstrate a continuous increase in RMSD or several rearrangement events. This behavior is further highlighted in Figure 2C, where the divergence from the average RMSD is plotted against the time-series average RMSD of the other Top6 replicas. The tight standard deviation of the mean shows how stable most of the simulations are and gives context to the magnitude of rearrangement in replicates 2 and 5. To investigate which portion of the TM domain of these replicas was contributing to the non-convergence, we performed further RMSD calculations on individual TM helices (Figure S1). This demonstrated a high RMSD in TM3 and TM5 for both replicas, which are known as the “greasy slide”, which is thought to be involved in both attracting substrate peptides bound to the C1 domain as well as ultimately in the release of substrate peptides into the bulk membrane.

**Figure 3:**
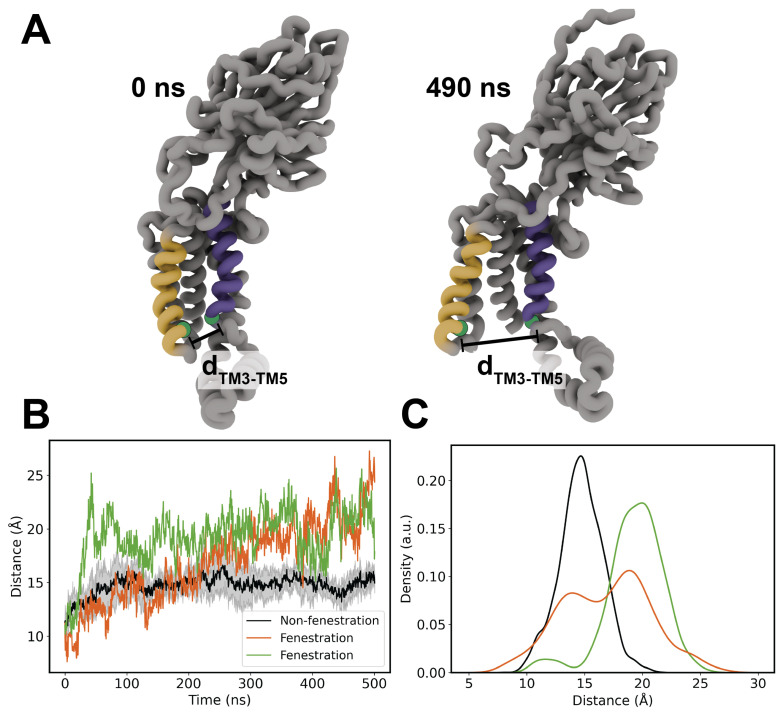
(**A**) Schematic of helical cracking distance measurement. The alpha carbons of G422 and Q491 are shown as green spheres at the lower half of TM3 and TM5, respectively. (**B**) Time series of helical distances of both cracking replicas against the bulk average of non-cracking replicas. (**C**) Helical distances binned by individual cracking replicas versus the aggregate data of non-cracking replicas.

To further probe the mobile TM3 and TM5 helices, we computed distance-over-time measurements of residues G422 and Q491, which are located at the bottom of TM5 and TM3, respectively (Figure 3A). This relationship was compared for Top6 replicas 2 and 5 versus the average of the other replicas just as with the RMSD calculation (Figure 3B). These data clearly resemble the RMSD comparison, suggesting that the source of the RMSD divergence is the cracking open of TM helices 3 and 5. We binned the data into histograms to more clearly demonstrate the shift in the helical distance distribution between cracking and non-cracking replicas (Figure 3C,D). The bimodality of this cracking distribution for replica 5 highlights the transient nature of the cracking and resealing of TM helices 3 and 5, which aligns nicely with the RMSD traces above.

We also compared the helical tilt angle of TM3/TM5 against the distance of G422 and Q491 to probe whether there was a relationship between TM tilt and the cracking phenomena (Figure S2). We defined the helical angle as the angle formed by the principal axis of each TM helix versus the bilayer normal, which was treated simply as a normal vector in the positive *z*-direction. There is no apparent contribution from the helical tilt angle, and we feel that the cracking distance is a sufficient descriptor of RMSD divergence in our systems. We compiled a representative movie of helical cracking from Top6 replica 2, which has been included in the Supplementary Material of this manuscript (Supplementary Material Movie S1).

### 3.3. Penetration of Full Lipids Cracks Open YidC

Findings of transmembrane helical cracking prompted a more in-depth qualitative inspection of the affected trajectories in order to identify whether these helices were merely more dynamic on their own or whether the local lipid environment had an effect on the TM structure of YidC. Interestingly, we observed penetration of an entire PE lipid in both of these simulation replicas. We have included a movie of the trajectory of this phenomena occurring in the Top6 replica 5 in the Supplementary Material (Supplementary Material Movie S2). In both replicas, the fenestrated lipid was a PMPE, but owing to the subtle differences in chemistry between the four PE lipid species sampled, we feel that this is explained by the fact that PMPE is the most abundant lipid in our membrane. In each case, the lipid is inserted in a headgroup-first fashion where the polar headgroup settles into the hydrophilic cavity of the YidC pore before the acyl tails enter, settling near the greasy TM3/TM5 slide. Hydrogen-bond analysis failed to identify any high-occupancy protein–lipid interactions, and it is our belief that while it is possible that low-occupancy hydrogen bonding may guide fenestration, the presence of water in the TM cavity may ultimately initiate full-lipid insertion due to the partial solvation of the TM pore.

Upon further inspection of other Top6 replicas and the coarser membrane compositions, we observe penetration of lipid acyl tails or headgroups in many of the systems, albeit not a full lipid molecule as in Top6 replicas 2 and 5. These partial lipid penetration events are likely responsible for the slight fluctuations we observe in RMSD of the other replicas, as well as the cracking distances reported for other systems (Figure S3). The density of full penetration events appears to be skewed towards the more realistic Top6 replicas, which may explain why this behavior was not observed in past simulation work. It is also our belief that because we generated more extensive sampling than other computational studies of YidC, we had a higher chance of capturing rare protein–lipid interactions such as full-lipid penetration.

**Figure 4:**
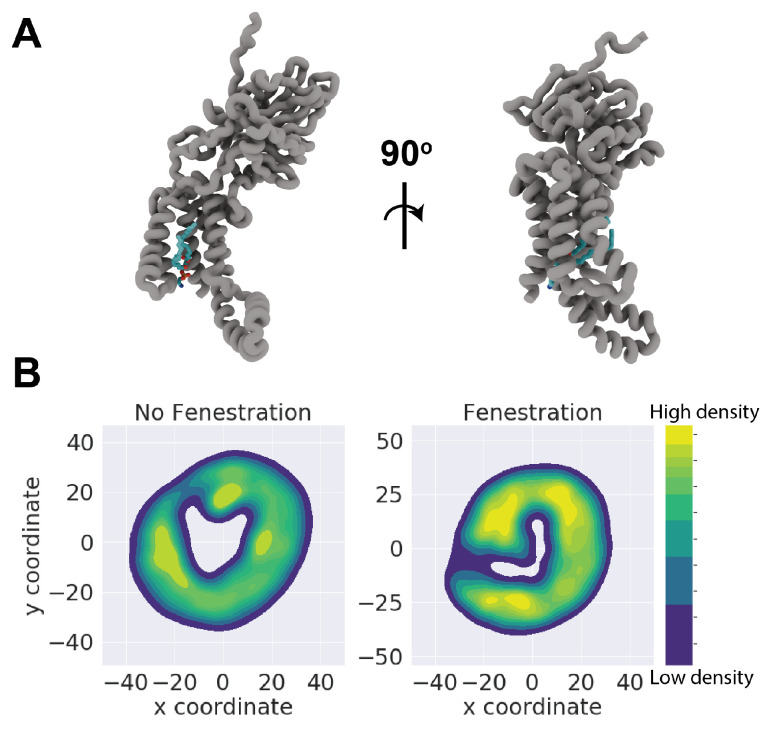
(**A**) Front and side views of full-lipid penetration from Top6 replica 5. Both the acyl tails and headgroup of PMPE residue 319 have fully inhabited the TM cavity of YidC. (**B**) Representative kernel density estimates for non-fenestrating replica 3 and fenestrating replica 5. KDE was performed on lipid phosphorous xy coordinates on lower leaflet lipid headgroups. Both KDEs are colorized on the same scale of arbitrary density units.

In order to provide a better estimation of lipid penetration, the distribution of lipid phosphorous atoms was used to generate a kernel density estimate. By binning xy coordinates of phosphorous atoms belonging to the cytoplasmic leaflet within 20 Å of the TM domain, we generated plots for both a model fenestrating and non-fenestrating simulation (replicas 5 and 3, respectively), shown in Figure 4. We chose to exclude lipids belonging to the periplasmic leaflet as we observed no penetration from these lipids, likely owing to TM cracking occurring only in the cytoplasmic end of the transmembrane region. To ensure that these plots are comparable, the protein was centered and aligned with the C1 loop pointing along the *y* axis throughout the trajectory. Patterns of residence in both systems appear to be somewhat consistent, but it is of note that the central cavity is unoccupied in the model non-fenestrating system. A key feature found in all replicas is a small indentation in the KDE plot, which indicates minor or transient lipid entry (Figure S4).

**Figure 5:**
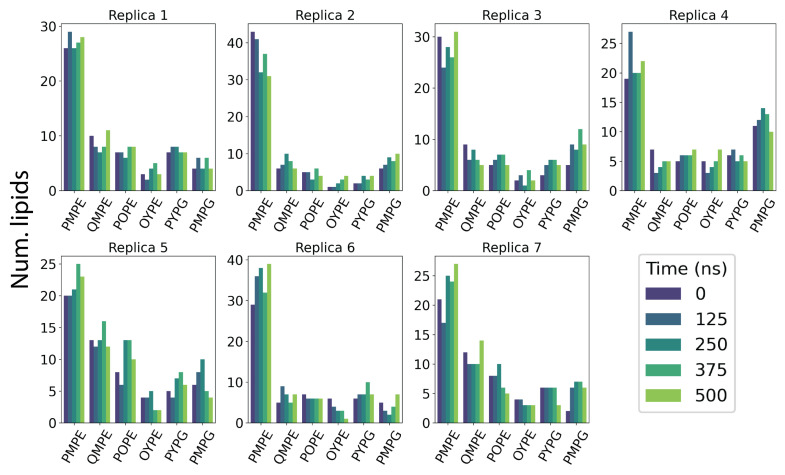
Lipid exchange in Top6 replicas. Lipid counts are computed at five simulation time points where lipids within a cutoff distance of 5 Å are considered local to the protein. Time points are taken at discrete 125 ns intervals throughout the simulation.

To ensure that the entry of lipids into the TM pore is not an artifact of local lipid composition, we analyzed the lipid distribution over 125 ns snapshots of each simulation (Figure 5). While the lipid composition is relatively dynamic in each replica and roughly approximates the bulk membrane distribution, there is no apparent correlation with local lipid identity. In simulations with transient or no lipid penetration events, we observe similar fluctuations in local lipid enrichment corroborating our findings that lipid penetration is independent of specific headgroup identity.

### 3.4. Dynamic Effects of Full-Lipid Penetration on YidC Fenestration

**Figure 6:**
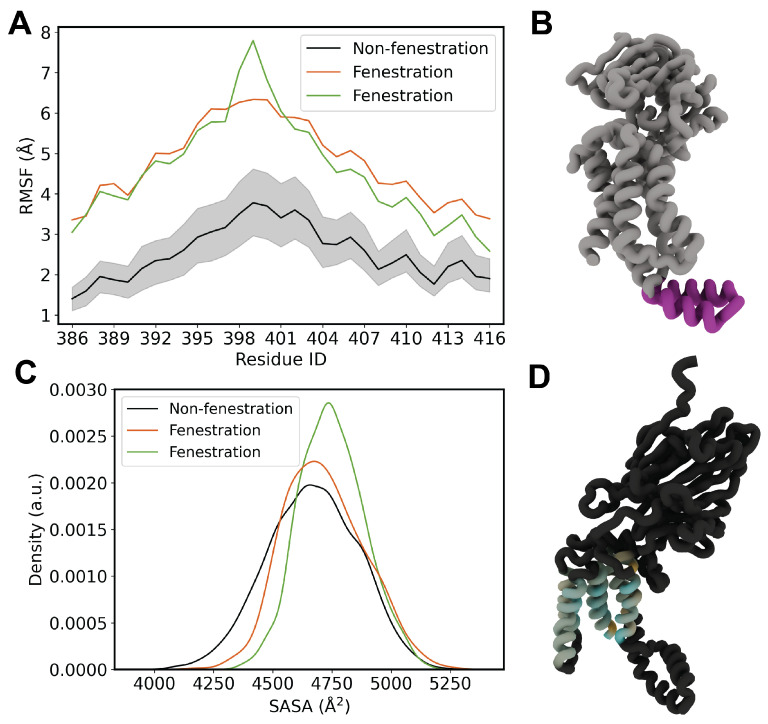
(**A**) RMSF of cytosolic C1 loop. (**B**) Molecular rendering of the C1 domain. (**C**) SASA distribution for fenestrating and non-fenestrating replicas. (**D**) Rendering of per-residue Δ SASA for TM residues between fenestrating replica 5 and non-fenestrating replicas.

In an effort to explore downstream effects of lipid penetration on other key regions of YidC, we turned our attention to the cytosolic C1 domain. We first performed root-mean square fluctuation (RMSF) calculations to assess any differences in flexibility as a result of lipid penetration (Figure 6). In Top6 replicas, which exhibit fenestration events, we observe a significant increase in the flexibility of the C1 domain, as much as a 100% increase for residues in the unstructured end of the coiled coil. This pattern of increased fluctuation did not appear to correlate for the rest of the protein, with the notable exception of TM5, which is expected based on the cracking behavior already reported (Supplementary Material Figure S5).

We finally computed the TM solvent-accessible surface area (SASA) for YidC in both the fenestrating and non-fenestrating systems (Figure 6C). We observe that for both fenestrating replicas, there is an increase in the SASA of the TM bundle. To better visualize this, we first computed the difference in mean SASA in a per-residue fashion for each system. We then applied the data to a molecular rendering in order to highlight hotspots of differential solvent accessibility (Figure 6D). This rendering demonstrates a marked increase in surface area for TM3 and TM6, which provide stabilization to both the hydrophobic and hydrophilic portions of substrate peptides, respectively.

## 4. Discussion

In this study, we employed molecular dynamics (MD) simulations to investigate the effect of protein–lipid interactions on both the transmembrane (TM) regions and membrane-adjacent domains of YidC, an important protein insertase in the *E. coli* inner membrane. Our simulations reveal key insights into how the surrounding lipid environment influences the structural and functional dynamics of YidC.

The robust equilibration of all simulated replicas, as indicated by the converged RMSD plots, supports the reliability of our results. Moreover, the agreement of bulk properties such as membrane thickness and deuterium order parameters with previously established membrane-only simulations by the Klauda group further validates our simulations [37,38]. The variability in the local lipid environment and lipid exchange between the annular layer and bulk membrane suggest reasonable sampling in our systems, ensuring that our analysis is likely to capture physiologically relevant protein–lipid interactions.

A primary function of YidC, particularly when complexed with the SecYEG machinery, is to assist in the insertion of larger membrane proteins [16]. Our observation of TM helices “cracking open” suggests a mechanism by which YidC facilitates substrate insertion, potentially providing an expanded refolding site for larger proteins. Membrane lipids are already implicated in the assembly of the SecYEG–YidC complex, and our findings suggest they may play a more active role, not only in structural support but also in preparing YidC for substrate interaction. The widening of the TM helices results in an increased cavity in the solvent-accessible surface area, which could enhance substrate engagement and accelerate binding kinetics.

**Figure 7:**
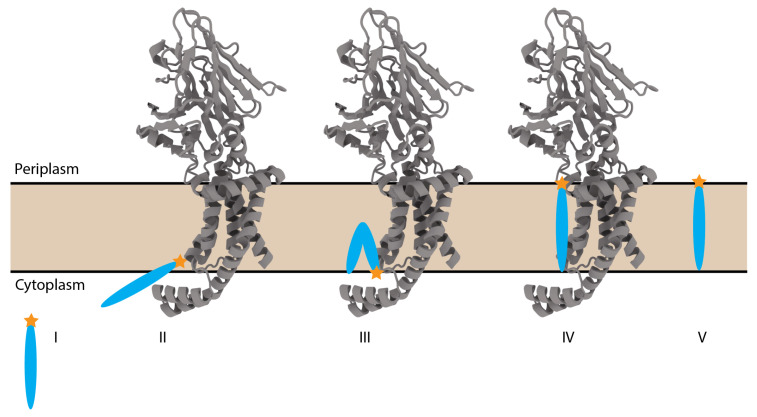
Representative schematic of the current mechanistic hypothesis of YidC insertion. Substrate peptide (I) is shown as a blue oval with the N-terminus shown as an orange star. The peptide is first recruited by the C1 domain (II), before folding in half as it begins to translocate up the transmembrane pore (III). The N-terminus then unfolds, crossing the span of the membrane (IV) before exiting YidC into the bulk membrane (V).

Our simulations also support the hypothesis that the hydrophobic residues along TM helices 3 and 5, which form the “greasy slide”, are critical for peptide loading [13]. Recent studies have shown that substrate peptides, such as the Pf3 coat protein, are inserted into YidC with their hydrophobic core first, stabilized by the hydrophobic interactions along TM3 and TM5 [14] (Figure 7). The penetrating lipids observed in our simulations indicate that protein–lipid interactions may directly contribute to this insertion mechanism. We propose that membrane phospholipids may participate in protein insertion by priming the TM pore and stabilizing substrate peptides through hydrophobic interactions. This lipid-mediated mechanism could facilitate the initial stages of substrate insertion, allowing for smooth peptide translocation before release into the membrane.

Additionally, the flexibility of the C1 domain, which is involved in substrate recruitment, was significantly increased in response to lipid fenestration. This aligns with previous work identifying the role of the C1 domain as a key player in ligand peptide recruitment [70]. The heightened flexibility observed in our simulations suggests that lipid interactions may enhance the mobility of the C1 domain, increasing its interaction frequency with substrate peptides. This supports a model where lipid-driven modulation of C1 dynamics facilitates the initial binding of substrate N-termini, leading to more frequent YidC-substrate interactions.

In summary, our findings suggest a role for protein–lipid interactions in regulating both the structural dynamics and functional activity of YidC. Lipids not only support the structural assembly of the SecYEG–YidC complex but may actively participate in substrate peptide insertion, by tuning either the thermodynamics or kinetics of binding. While outside the scope of this preliminary exploration, this hypothesis could be probed by further simulation work in which the insertion of a substrate peptide is examined in both the non-fenestrating and fenestrating contexts. Although these interactions are difficult to probe experimentally due to the dynamic nature of lipid binding, our simulations provide a potential mechanism by which the membrane contributes to the functional regulation of YidC, extending beyond its traditional role as a passive structural element.

## Figures and Tables

**Table 1:** Lipid diversity of Top6 replicas.

Lipid	POPE	QMPE	PMPE	OYPE	PMPG	PYPG
Upper Leaflet	40	40	148	24	32	28
Lower Leaflet	40	40	156	24	32	28

## Data Availability

Data are available upon request from the authors.

## Supplementary Materials

The following supporting information can be downloaded at: https://www.mdpi.com/article/10.3390/membranes14120249/s1.
