# Supplementary material for: The Role of Protein–Lipid Interactions in Priming the Bacterial Translocon
Source: Membranes (Basel). 2024 Nov 24;14(12):249. doi: 10.3390/membranes14120249 (PMC11677795; doi:10.3390/membranes14120249)
Supplement: Supplementary file 1 [file membranes-14-00249-s001.zip › si-figures.pdf]

Supplementary Information

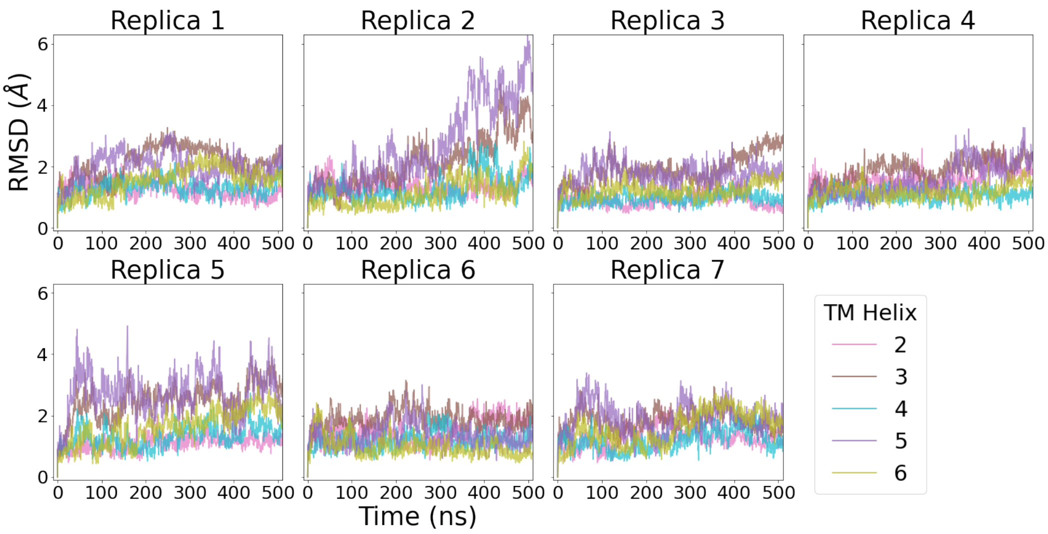

Figure S1. RMSD of each TM helix in Top6 replicas. TM3 and TM5 demonstrate strong divergence from the bulk RMSD value of 1.5 Å, with a maximal RMSD of 6 Å for TM5 in Top6 replica 2.

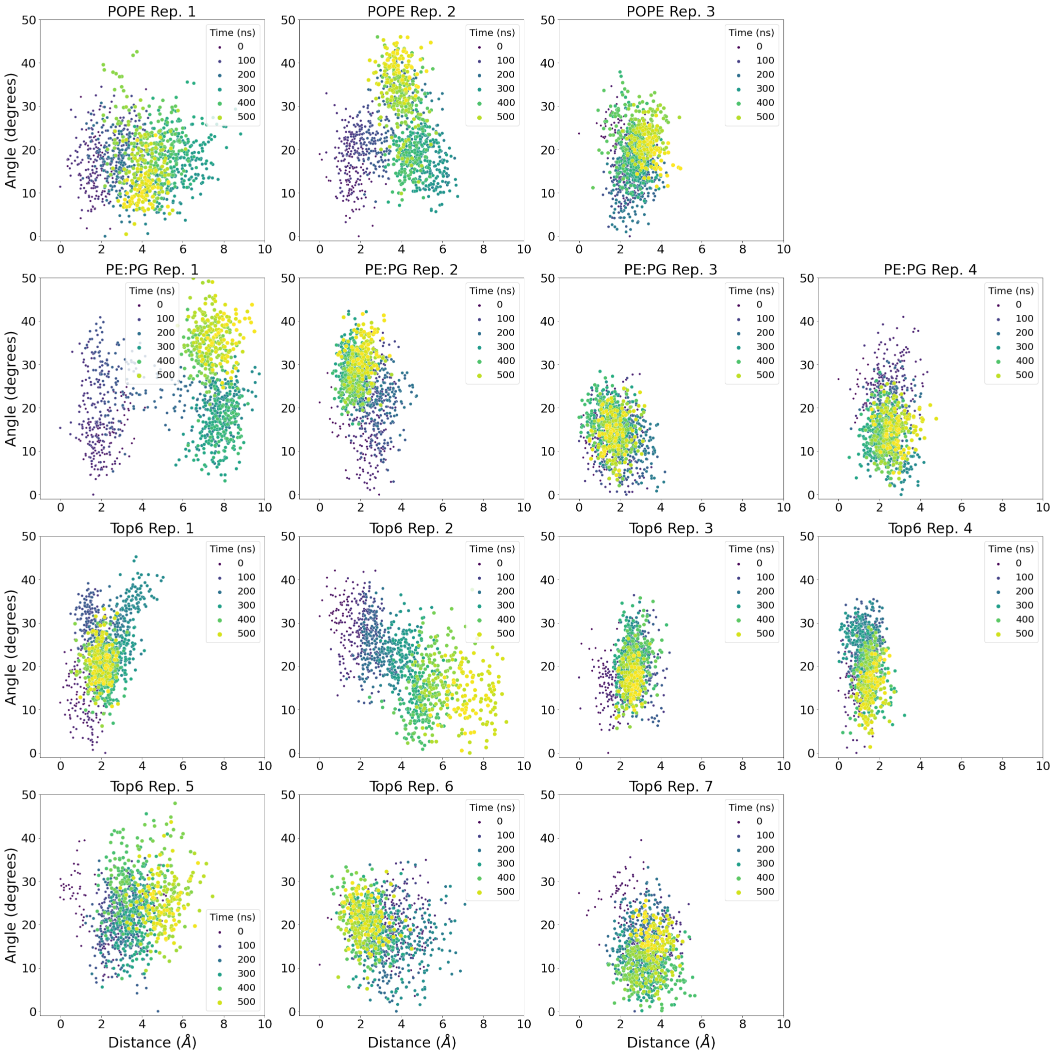

Figure S2. Plots of cracking distance versus angle of TM5 and TM3. There appears to be little correlation between transmembrane helix angle and fenestration relative to helical distance.

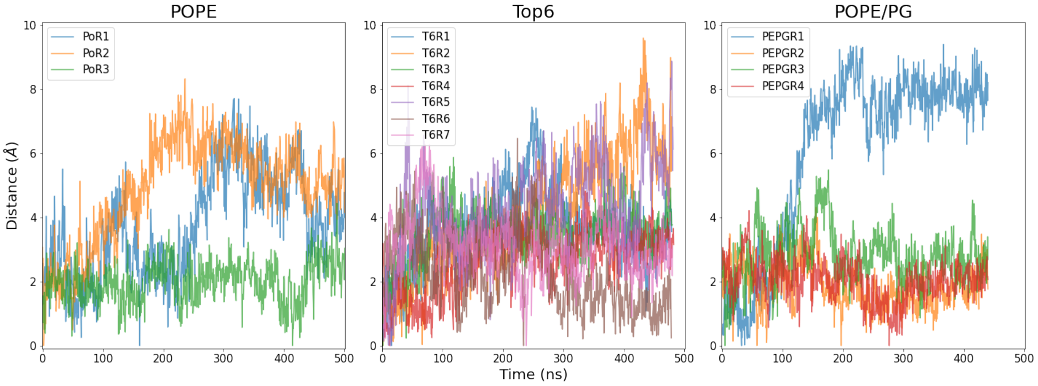

Figure S3. Raw helical cracking distances. There is clear, but transient, cracking open of TM helices in POPE replicas 1 and 2 as well as long-time opening in POPE:POPG replica 1.

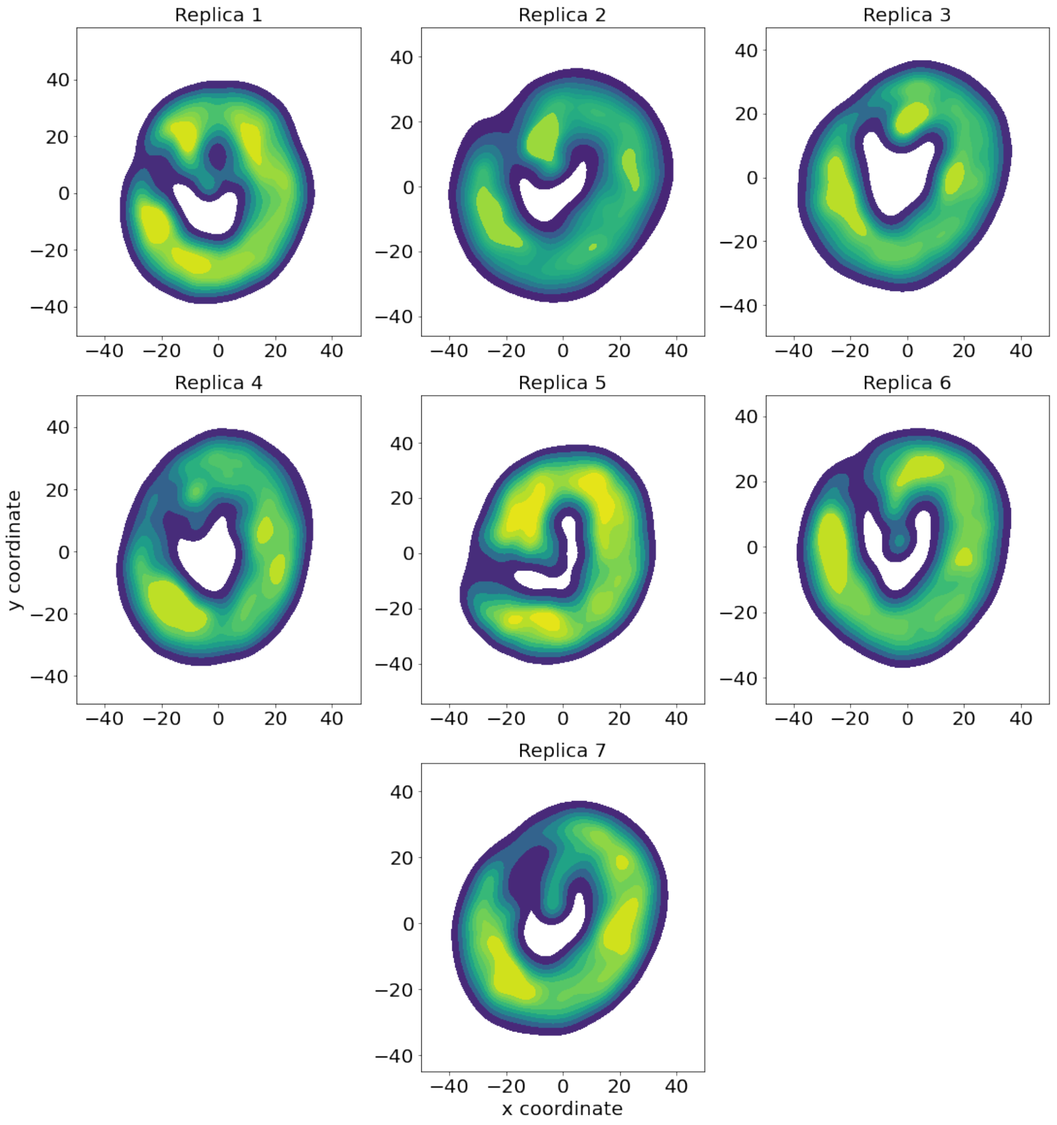

Figure S4. Kernel density estimates of Top6 replicas. Despite full-lipid penetration occurring only in replicas 2 and 5, there is clear density in the transmembrane space in several other replicas, notably replicas 1, 6, and 7.

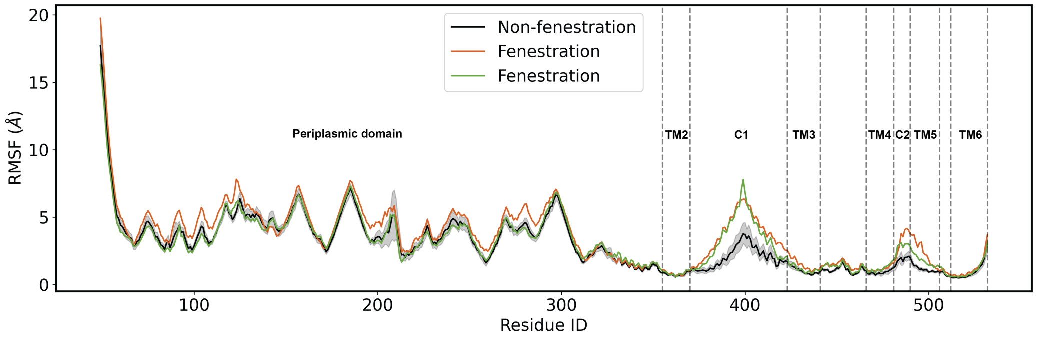

Figure S5. Comparison of C-alpha RMSF for all residues in fenestrating and non-fenestrating simulations. Structural regions are noted including the TMs 2-6, periplasmic domain, C1 domain and C2 loop which sits in between TM4/TM5. Fluctuations in periplasmic domain do not appear to completely correlate with fenestration.
